# Effect of adjuvant radiotherapy on overall survival and breast cancer-specific survival of patients with malignant phyllodes tumor of the breast in different age groups: a retrospective observational study based on SEER

**DOI:** 10.1186/s13014-024-02442-5

**Published:** 2024-05-21

**Authors:** Ping Yang, Gongyin Zhang, Yu Zhang, Wanying Zhao, Jinhai Tang, Siyuan Zeng, Xiupeng Lv, Li Lv

**Affiliations:** 1 Department of Radiation Oncology, The First Affiliated Hospital of Dalian Medical University, Dalian, Liaoning China; 2 Department of Breast and Hernia Surgery, The First Affiliated Hospital of Nanchang University, Nanchang, Jiangxi China; 3 Dalian Municipal Central Hospital, China Medical University, Shenyang, Liaoning China; 4 Department of Pathology, The Second Affiliated Hospital of Dalian Medical University, Dalian, Liaoning China

**Keywords:** Malignant phyllodes tumors, Radiotherapy, Overall survival (OS), Breast cancer-specific survival (BCSS)

## Abstract

**Purpose:**

Malignant phyllodes tumor of the breast (MPTB) is a rare type of breast cancer, with an incidence of less than 1%. The value of adjuvant radiotherapy (RT) for MPTB has been controversial. The aim of the study was to explore the effect of radiotherapy on the long-term survival of female patients with MPTB at different ages.

**Methods:**

Female MPTB patients were selected from the Surveillance, Epidemiology, and End Results (SEER) database between 2000 and 2020. A Kaplan–Meier survival analysis was conducted to investigate the value of RT for the long-term survival of MPTB patients in different age groups. Additionally, univariate and multivariate Cox regression analyses were performed for overall survival (OS) and breast cancer-specific survival (BCSS) of MPTB patients. Furthermore, propensity score matching (PSM) was also performed to balance the differences in baseline characteristics.

**Results:**

2261 MPTB patients were included in this study, including 455 patients (20.12%) with RT and 1806 patients (79.88%) without RT. These patients were divided into four cohorts based on their ages: 18–45, 46–55, 56–65, and 65–80. Before adjustment, there was a statistically significant difference in long-term survival between RT-treated and non-RT-treated patients in the younger age groups (age group of 18–45 years: OS *P* = 0.019, BCSS *P* = 0.016; age group of 46–55 years: OS *P* < 0.001, BCSS *P* < 0.001). After PSM, no difference was found in long-term survival of patients in both younger and older groups regardless of whether they received RT (age group of 18–45 years: OS *P* = 0.473, BCSS *P* = 0.750; age group of 46–55 years: OS *P* = 0.380, BCSS *P* = 0.816, age group of 56–65 years: OS *P* = 0.484, BCSS *P* = 0.290; age group of 66–80 years: OS *P* = 0.997, BCSS *P* = 0.763). In multivariate COX regression analysis, RT did not affect long-term survival in patients with MPTB.

**Conclusion:**

There is no evidence that long-term survival of MPTB patients in specific age groups can benefit from RT.

**Supplementary Information:**

The online version contains supplementary material available at 10.1186/s13014-024-02442-5.

## Introduction

Phyllodes tumor of the breast (PTB) is a rare disease that occurs predominantly in women, with an incidence ranging from 0.3 to 0.9% of all breast tumors and 2–3% of fibroepithelial tumors [1]. Lobular tumors are divided into benign, borderline, or malignant based on tumor margins, mesenchymal overgrowth, tumor necrosis and cellular anisotropy [2]. Of these, malignant phyllodes tumor of the breast (MPTB) is notorious for its recurrence and distant metastasis rates. Surgery is the mainstay of treatment for phyllode tumors, including mastectomy and breast-conserving surgery. Despite surgical intervention, the local recurrence rate remains as high as 65% [3, 4]. In addition, the distant metastasis rate of MPTB can be up to 20–30% [5]. Compared with lymph node metastasis, hematogenous metastasis is the main way of MPTB metastasis, and the lung, bone and abdominal viscera are the most common sites of distant metastasis [6]. The value of postoperative adjuvant treatments for MPTB, such as radiotherapy (RT), has also been controversial. There are insufficient data from prospective studies on radiotherapy for the treatment of PTB [7]. A previous study concluded that patients with malignant lobular tumors who received adjuvant radiotherapy had worse survival outcomes compared to those who did not receive adjuvant radiotherapy [8]. Another study found that women with MPTB who received adjuvant radiotherapy after surgery had a significantly reduced local recurrence, but no improvement in disease-free survival (DFS) and overall survival (OS) [9]. Our previous study found that adjuvant radiotherapy for patients with stage T3 or T4 MPTB did not affect OS or BCSS [10]. Furthermore, a study analyzed the effect of postoperative adjuvant radiotherapy on the long-term survival of MPTB patients diagnosed with T3N0M0 in different age groups. It was found that radiotherapy improved the survival of older patients, especially those over 65, while there was no significant benefit in younger patients with T3N0M0 [11]. As a result, the role of RT in MPTB remains unclear, despite its increasing use.

SEER database was utilized in this study to explore the value of adjuvant radiotherapy for long-term survival of MPTB patients of different ages. The results showed that MPTB patients did not benefit from adjuvant radiotherapy.

## Methods

### Data sources

The clinical data of MPTB patients were obtained from the SEER database.

### Study population

**Fig. 1:**
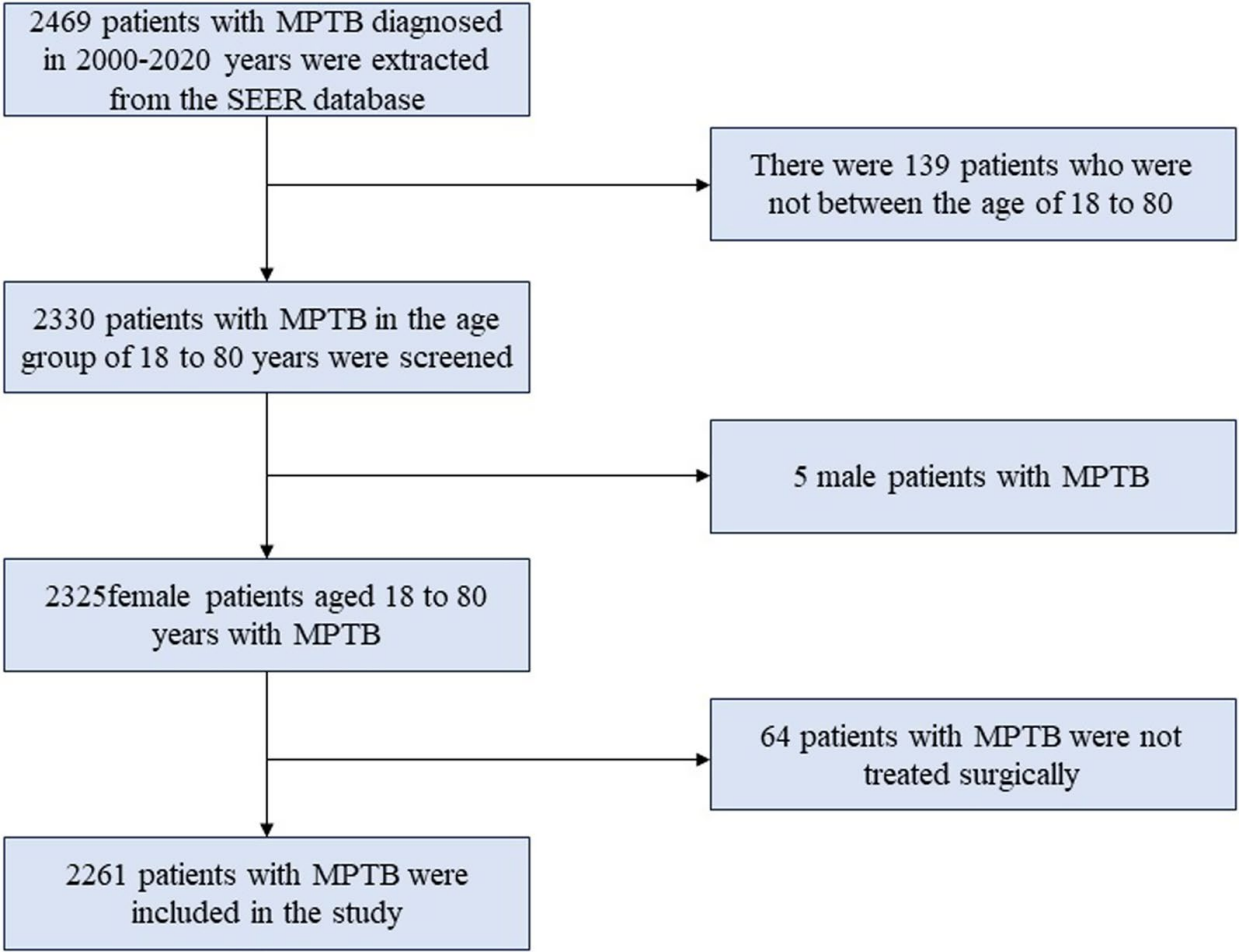
Detailed data collection process

The information on MPTB patients was collected from the SEER database between 2000 and 2020. Inclusion criteria for MPTB patients: female with MPTB (ICD-O-39020/3), diagnosed between 2000 and 2020. MPTB patients who did not undergo surgery and male MPTB patients were excluded. The radiotherapy, chemotherapy, age at diagnosis, local lymphatic biopsy, surgery of primary site, race, death status, long-term survival, tumor grade, marital status, laterality, distant metastatic status, T stage and lymph node status were extracted from the SEER database. Finally, 2261 patients with MPTB were included in the study, including 455 patients (20.12%) with RT and 1806 patients (79.88%) without RT. These patients were divided into four groups based on their age: 18–45, 46–55, 56–65, and 66–80 years. More detailed information about the screening process is presented in Fig. 1.

### Statistical analysis

The long-term survival outcomes in this study were assessed by the overall survival (OS) and breast cancer-specific survival (BCSS), respectively [12]. OS referred to the time from tumor diagnosis to death from any cause, while BCSS denoted the time from tumor diagnosis to death from breast cancer [13].

Kaplan–Meier survival estimation was performed to compare the differences in OS and BCSS between different age groups, and the log-rank test was used to assess the Kaplan–Meier curve. Comparisons between groups of categorical variables were performed using the Chisq test and Yates’ correction for continuity. The effect of age on adjuvant RT was examined using the COX analysis and the propensity score matching (PSM) method [14, 15]. The effect of adjuvant RT on MPTB patients in different age groups was analyzed by the above methods. R 4.2.2 software and IBM SPSS Statistics 27.0 were used for statistical analysis, with a *p*-value < 0.05 as the threshold of statistical significance.

## Results

### Clinical and demographic characteristics of patients

The demographic and clinical characteristics of all 2261 patients are summarized in Table 1 by age group. The patients were divided into four cohorts based on their ages: 18–45, 46–55, 56–65, and 65–80. In the four groups, there are significant differences (*P* < 0.05) in race, tumor grade, lymph node status, RT, marital status, and local lymphatic biopsy. According to the baseline characteristics of patients with MPTB, the value of RT could be discussed in different cohorts based on different clinicopathological characteristics.

**Table 1:** Clinical characteristics of MPTB patients diagnosed in 2000–2020 from the SEER database

Characteristics	18–45	46–55	56–65	66–80	P value
N	729	769	489	274	
Year					0.614
2000–2009	347 (47.6%)	381 (49.5%)	225 (46%)	127 (46.4%)	
2010–2020	382 (52.4%)	388 (50.5%)	264 (54%)	147 (53.6%)	
Race					< 0.001
White	483 (66.3%)	546 (71%)	366 (74.8%)	220 (80.3%)	
Black	98 (13.4%)	82 (10.7%)	42 (8.6%)	21 (7.7%)	
Other	148 (20.3%)	141 (18.3%)	81 (16.6%)	33 (12%)	
Tumor grade					< 0.001
Unknown	301 (41.3%)	164 (21.3%)	131 (26.8%)	45 (16.4%)	
I–II	267 (36.6%)	379 (49.3%)	232 (47.4%)	151 (55.1%)	
III–IV	161 (22.1%)	226 (29.4%)	126 (25.8%)	78 (28.5%)	
Laterality					0.322
Left	346 (47.5%)	385 (50.1%)	253 (51.7%)	126 (46%)	
Right	383 (52.5%)	384 (49.9%)	236 (48.3%)	148 (54%)	
AJCC.T					0.591
Unknown	123 (16.9%)	107 (13.9%)	70 (14.3%)	44 (16.1%)	
T1–T2	342 (46.9%)	357 (46.4%)	227 (46.4%)	133 (48.5%)	
T3–T4	264 (36.2%)	305 (39.7%)	192 (39.3%)	97 (35.4%)	
AJCC.N					< 0.001
Unknown	100 (13.7%)	46 (6%)	25 (5.1%)	11 (4%)	
Negative	604 (82.9%)	717 (93.2%)	453 (92.6%)	257 (93.8%)	
Positive	25 (3.4%)	6 (0.8%)	11 (2.2%)	6 (2.2%)	
AJCC.M					0.979
Unknown	101 (13.9%)	110 (14.3%)	69 (14.1%)	44 (16.1%)	
Negative	616 (84.5%)	645 (83.9%)	413 (84.5%)	225 (82.1%)	
Positive	12 (1.6%)	14 (1.8%)	7 (1.4%)	5 (1.8%)	
Surgery of primary site					0.323
Breast-conserving surgery	404 (55.4%)	408 (53.1%)	259 (53%)	134 (48.9%)	
Mastectomy	325 (44.6%)	361 (46.9%)	230 (47%)	140 (51.1%)	
Radiotherapy					0.020
No	597 (81.9%)	617 (80.2%)	367 (75.1%)	225 (82.1%)	
Yes	132 (18.1%)	152 (19.8%)	122 (24.9%)	49 (17.9%)	
Chemotherapy					0.675
No	695 (95.3%)	735 (95.6%)	468 (95.7%)	266 (97.1%)	
Yes	34 (4.7%)	34 (4.4%)	21 (4.3%)	8 (2.9%)	
Marital status					< 0.001
Unknown	56 (7.7%)	47 (6.1%)	30 (6.1%)	11 (4%)	
Married	372 (51%)	450 (58.5%)	273 (55.8%)	126 (46%)	
Not married	301 (41.3%)	272 (35.4%)	186 (38%)	137 (50%)	
Local lymphatic biopsy					0.009
Yes	149 (20.4%)	194 (25.2%)	130 (26.6%)	81 (29.6%)	
No	580 (79.6%)	575 (74.8%)	359 (73.4%)	193 (70.4%)	

### Survival analysis of MPTB patients treated and untreated with adjuvant radiotherapy

**Fig. 2:**
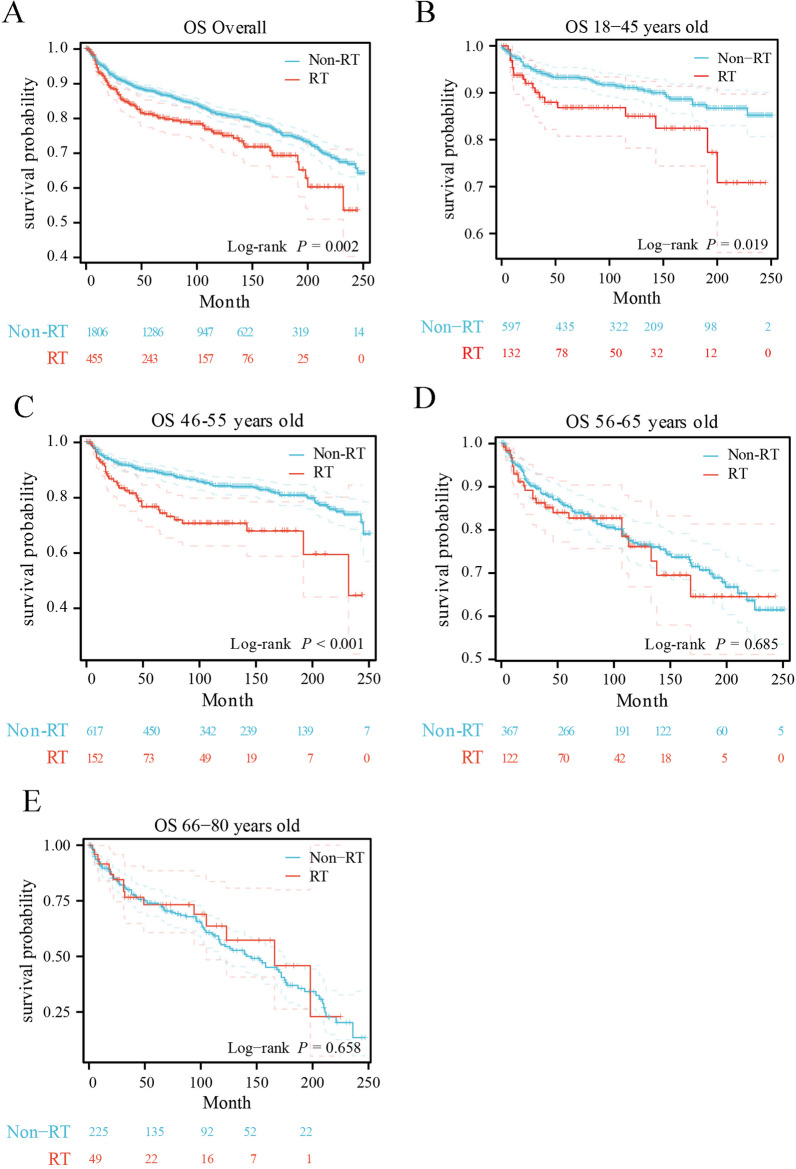
Kaplan–Meier overall survival curves of MPTB patients in different age groups based on the RT status. **A** Overall, **B** 18–45 years, **C** 46–55 years, **D** 56–65 years, **E** 66–80 years

**Fig. 3:**
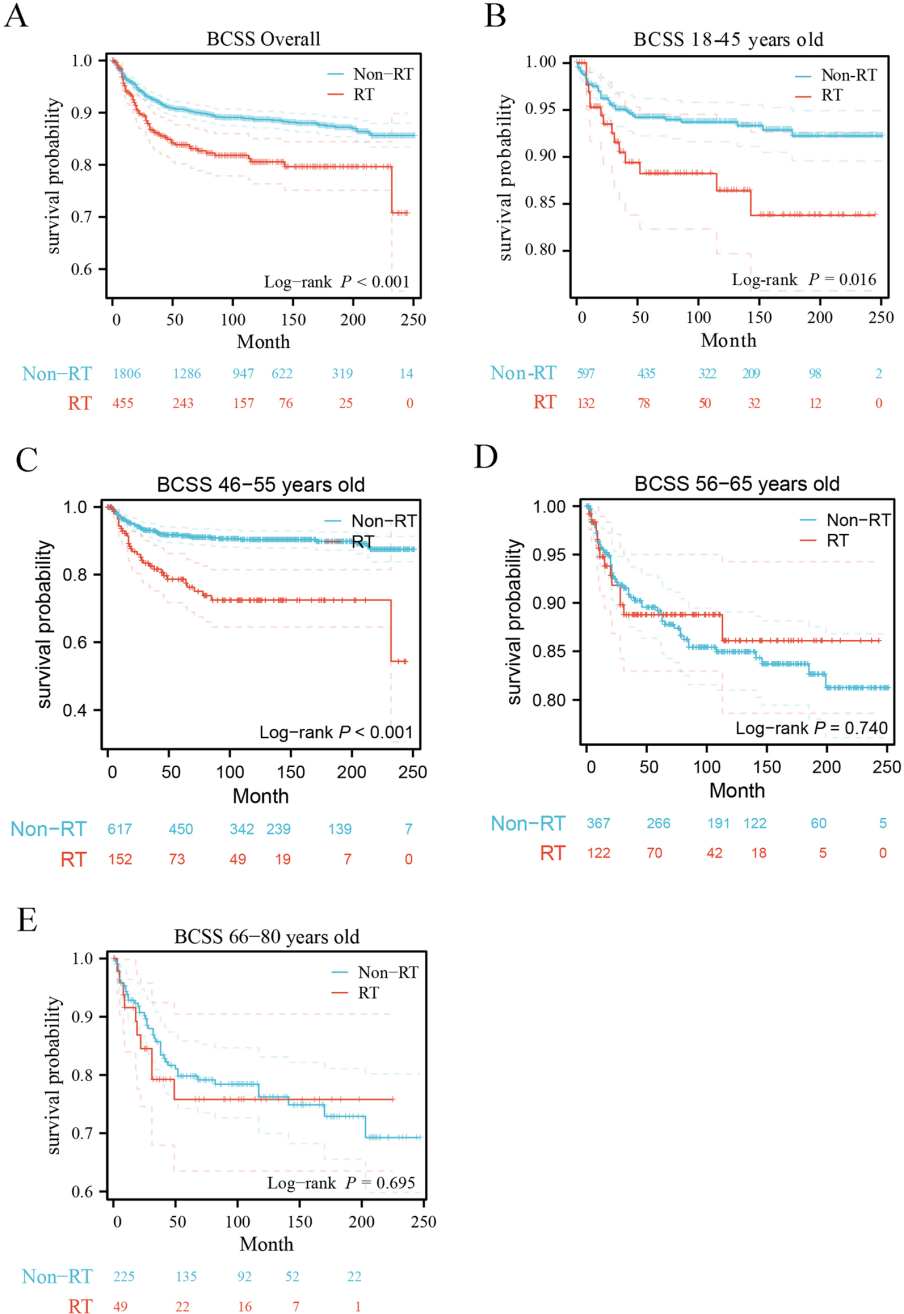
Kaplan–Meier breast cancer-specific survival curves of MPTB patients in different age groups based on the RT status. **A** Overall, **B** 18–45 years, **C** 46–55 years, **D** 56–65 years, **E** 66–80 years

The OS and BCSS of MPTB patients treated and untreated with RT were evaluated by the Kaplan–Meier (K–M) survival curves. Compared to patients treated with RT, patients untreated with RT had better OS (*P* = 0.002) (Fig. 2A) and BCSS (*P* < 0.001) (Fig. 3A). K–M survival curves revealed there was no statistically significant difference in survival between patients in the age group of 56–65 years and patients in the age group of 66–80 years treated and untreated with RT (age group of 56–65 years: OS *P* = 0.685, BCSS *P* = 0.740; age group of 66–80 years: OS *P* = 0.658, BCSS *P* = 0.695, respectively). However, for OS and BCSS, there was a statistically significant difference between patients who received RT and patients who did not receive RT in the age groups of 18–45 years and 46–55 years (age group of 18–45 years: OS *P* = 0.019, BCSS *P* = 0.016; age group of 46–55 years: OS *P* < 0.001, BCSS *P* < 0.001, respectively), and the patients untreated with RT had significant better long-term survival outcomes compared to patients treated with RT. Detailed information on survival analysis results is shown in Figs. 2B–E and 3B–E.

### Univariate and multivariate COX regression analyses of different variables

Considering that patients with MPTB of different ages exhibit distinct clinicopathological characteristics, univariate and multivariate Cox regression models were used to assess the effects of variables in four different age groups. After balancing the effects of other factors, as shown in Table 2 and Additional file 1: Table S1, no patients with MPTB were found to be able to benefit from RT, either in younger (age group of 18–45 years: OS HR = 0.694, 95% CI 0.365–1.317, *P* = 0.263; BCSS HR = 1.097, 95% CI 0.504–2.386, *P* = 0.816; age group of 46–55 years: OS HR = 0.972, 95% CI 0.635–1.486, *P* = 0.894, BCSS HR = 1.294, 95% CI 0.748–2.241, *P* = 0.357, respectively) or older age groups (age group of 56–65 years: OS HR = 1.076, 95%CI: 0.827—1.401, *P* = 0.585, BCSS HR = 0.633, 95% CI 0.319–1.258, *P* = 0.192; age group of 66–80 years: OS HR = 0.659, 95% CI 0.377–1.151, *P* = 0.143, BCSS HR = 1.024, 95% CI 0.483–2.170, *P* = 0.951, respectively).

**Table 2:** Multivariate Cox regression model analysis of OS in different age groups

Charactreristics	18–45		46–55		56–65		66–80	
	HR (95%C)	*P*	HR (95%C)\	*P*	HR (95%C)	*P*	HR (95%C)	*P*
Year								
2000–2009	Reference		Reference		Reference		Reference	
2010–2020	1.961 (1.110–3.466)	0.020	1.263 (0.830–1.923)	0.275	229.423 (78.300–672.223)	< 0.001	1.121 (0.702–1.790)	0.632
Race								
White	Reference		Reference		Reference		Reference	
Black	1.367 (0.695–2.690)	0.365	1.507 (0.933–2.434)	0.094	0.894 (0.5981.336)	0.583	0.763 (0.324–1.797)	0.535
Other	1.170 (0.641–2.137)	0.609	1.062 (0.642–1.757)	0.815	1.412 (1.061–1.878)	0.018	0.430 (0.195–0.947)	0.636
Tumor grade								
I–II	Reference		Reference		Reference		Reference	
III–IV	5.588 (2.346–13.309)	< 0.001	3.963 (2.591–6.060)	< 0.001	0.960 (0.729–1.264)	0.769	1.481 (0.964–2.276)	0.073
Unknown	3.126 (1.302–7.507)	0.011	1.300 (0.732–2.308)	0.371	1.256 (0.976–1.617)	0.077	1.623 (0.936–2.814)	0.085
Laterality								
Left	Reference		Reference		Reference		Reference	
Right	1.192 (0.713–1.991)	0.503	1.298 (0.922–1.828)	0.134	1.097 (0.889–1.355)	0.387	0.621 (0.425–0.906)	0.014
AJCC.T								
T1–T2	Reference		Reference		Reference		Reference	
T3–T4	2.566 (1.369–4.809)	0.003	2.753 (1.818–4.169)	< 0.001	1.389 (1.077–1.792)	0.011	2.447 (1.557–3.846)	< 0.001
Unknown	0.188 (0.034–1.027)	0.054	1.370 (0.309–6.066)	0.679	Inf	0.992	1.928 (0.741–5.017)	0.179
AJCC.N								
Negative	Reference		Reference		Reference		Reference	
Positive	5.500 (2.560–11.815)	< 0.001	1.874 (0.559–6.281)	0.308	0.524 (0.165–1.666)	0.274	3.892 (1.320–11.477)	0.014
Unknown	0.522 (0.058–4.726)	0.563	0.849 (0.430—1.676)	0.637	0.400 (0.223–0.719)	0.002	0.800 (0.338—1.893)	0.612
AJCC.M								
Negative	Reference		Reference		Reference		Reference	
Positive	8.925 (3.063–26.002)	< 0.001	6.722 (3.007–15.024)	< 0.001	0.000 (0.000-Inf)	0.994	NA	
Unknown	50.545 (3.477–734.730)	0.004	3.083 (0.750–12.676)	0.119	0.000 (0.000-Inf)	0.994	NA	
Surgery of primary site								
BCS	Reference		Reference		Reference		Reference	
Mastectomy	2.258 (1.213–4.202)	0.010	1.360 (0.917–2.018)	0.126	0.768 (0.602–0.981)	0.034	0.933 (0.601–1.448)	0.758
Radiotherapy								
No	Reference		Reference		Reference		Reference	
Yes	0.694 (0.365–1.317)	0.263	0.972 (0.635–1.486)	0.894	1.076 (0.827–1.401)	0.585	0.659 (0.377–1.151)	0.143
Chemotherapy								
No	Reference		Reference		Reference		Reference	
Yes	1.658 (0.719–3.826)	0.236	2.701 (1.468–4.968)	0.001	1.130 (0.640–1.996)	0.672	3.561 (1.511–8.391)	0.004
Marital status								
Married	Reference		Reference		Reference		Reference	
Unmarried	1.143 (0.651–2.006)	0.642	1.167 (0.807–1.687)	0.411	1.236 (0.980–1.559)	0.074	2.051 (1.399–3.009)	** < 0.001**
Unknown	2.449 (1.008–5.949)	0.048	0.842 (0.371–1.910)	0.681	0.893 (0.583–1.368)	0.602	3.855 (1.673–8.882)	**0.002**
Local-lymphatic biopsy								
No	Reference		Reference		Reference		Reference	
Yes	0.741 (0.431–1.276)	0.280	0.937 (0.632–1.388)	0.746	1.167 (0.892–1.526)	0.261	0.768 (0.504–1.169)	0.218

### Survival analysis after propensity score matching

**Fig. 4:**
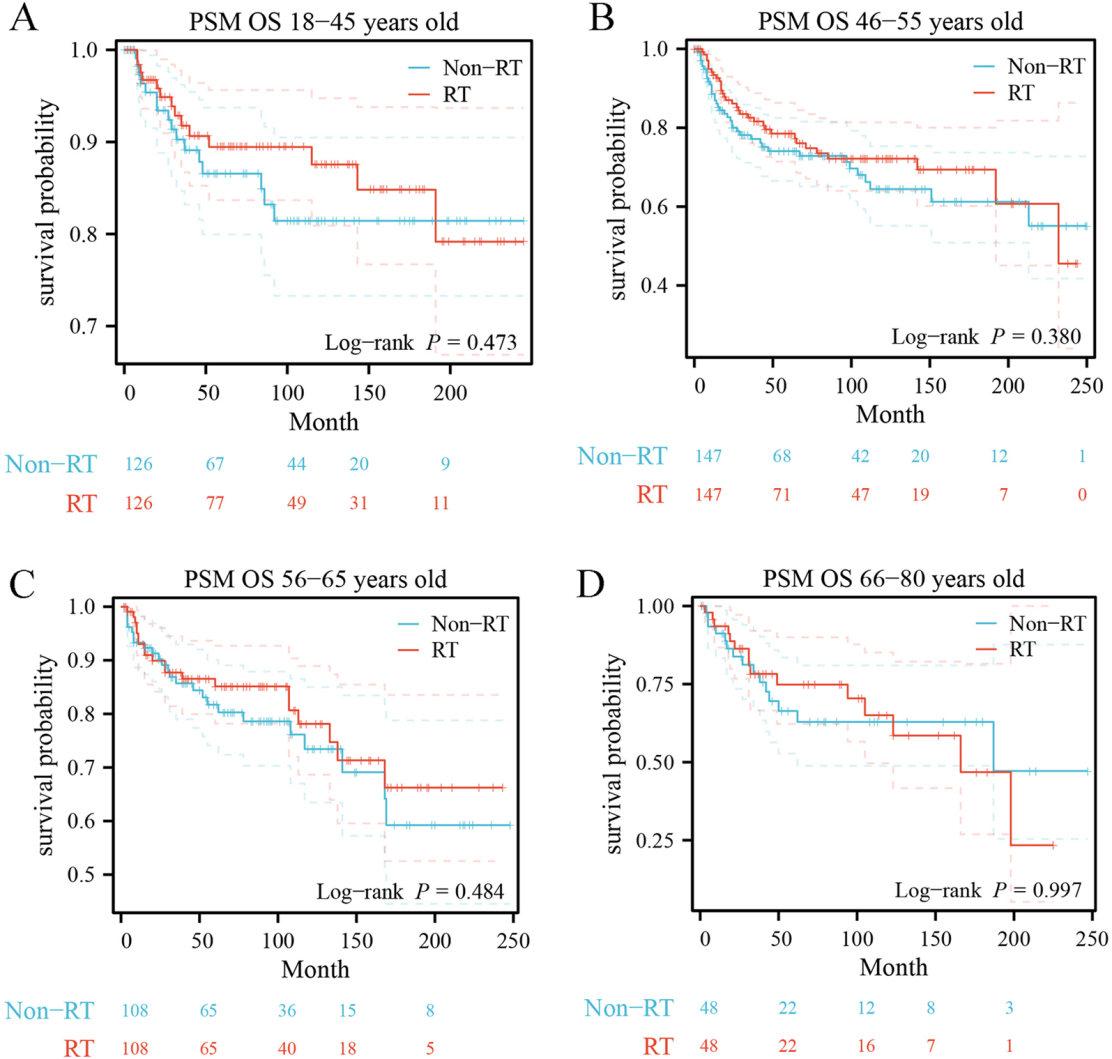
Kaplan–Meier survival estimates of overall survival comparing 1:1 matched Without vs With RT in different age groups. **A** 18–45 years, **B** 46–55 years, **C** 56–65 years, **D** 66–80 years

**Fig. 5:**
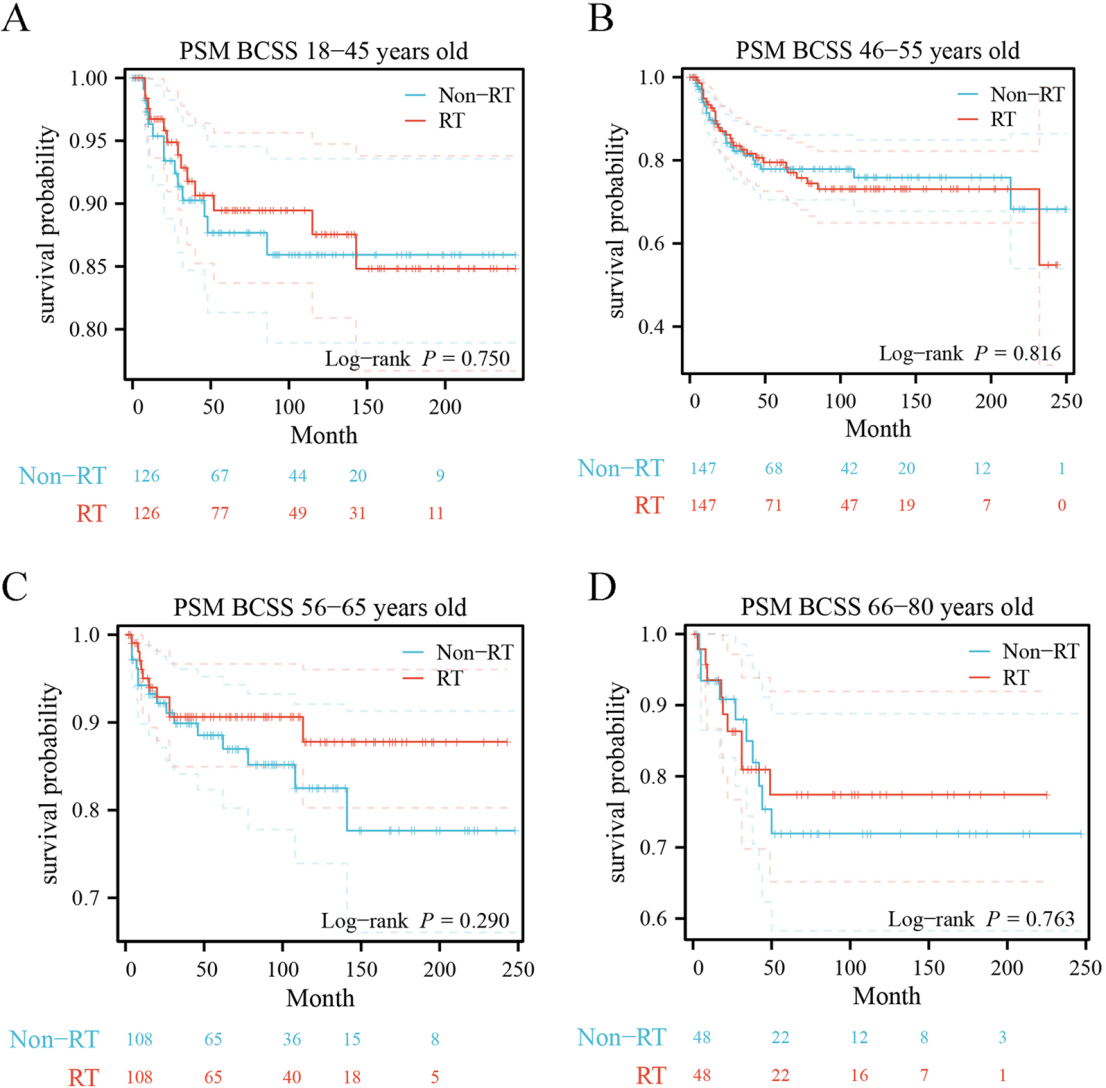
Kaplan–Meier survival estimates of breast cancer-specific survival comparing 1:1 matched Without vs With RT in different age groups. **A** 18–45 years, **B** 46–55 years, **C** 56–65 years, **D** 66–80 years

To address variations in baseline characteristics across the four cohorts and minimize bias attributed to other variables, a 1:1 case–control analysis was executed for comparing patients who received radiation therapy (RT) with those who did not receive RT through propensity score matching (PSM). After PSM, eleven factors were enrolled, including the year of diagnosis, race, marital status, laterality, tumor grade, T stage, lymph node status, distant metastatic status, surgery of primary site, local lymphatic biopsy, and chemotherapy (Additional file 2: Table S2). After PSM, no difference in long-term survival outcomes was found in the younger group (age group of 18–45 years: OS *P* = 0.473, BCSS *P* = 0.750; age group of 46–55 years: OS *P* = 0.380, BCSS *P* = 0.816, respectively) (Figs. 4A, B and 5A, B), although patients untreated with RT before PSM had better long-term survival outcomes than those treated with RT (age group of 18–45 years: OS *P* = 0.019, BCSS *P* = 0.016; age group of 46–55 years: OS *P* < 0.001, BCSS *P* < 0.001, respectively). Similarly, in the older age groups, there was still no significant difference between patients treated with RT and patients untreated with RT (age group of 56–65 years: OS *P* = 0.484, BCSS *P* = 0.290; age group of 66–80 years: OS *P* = 0.997, BCSS *P* = 0.763, respectively) (Figs. 4C, D and 5C, D), which is consistent with the pre-PSM results.

## Discussion

The value of RT for MPTB has been controversial for nearly 20 years. An analysis indicated that adjuvant RT reduced local recurrence in MPTB patients who underwent breast conservation surgery [9]. However, a previous study showed that adjuvant RT reduced local recurrence in borderline and malignant phyllode tumors without affecting disease-free survival or overall survival [16], while another study showed that adjuvant radiotherapy for malignant phyllodes tumor of the breast was only beneficial in reducing local recurrence-free survival (LRFS) [17]. In addition, a retrospective cohort study found that patients with MPTB > 5 cm in size who received breast-conserving surgery followed by adjuvant radiotherapy exhibited prolonged disease-specific and overall survival [18]. The results of a meta-analysis showed that adjuvant radiotherapy could reduce local recurrence, especially in MPTB patients aged < 45 years with tumor size > 5 cm [19]. The NCCN guidelines do not recommend postoperative adjuvant radiotherapy for MPTB [20], but the European Society of Medical Oncology (ESMO) does recommend postoperative adjuvant radiotherapy for low-grade soft-tissue sarcomas or tumors > 5 cm in size [21]. The adjuvant radiotherapy dose is mostly 50.0 Gy/2 Gy/25F [17, 22], and the tumor bed (TB) dose can be increased to 66 Gy [23].

One study showed that MPTB patients with more adverse prognostic factors received postoperative radiotherapy, but there was no statistically significant difference in BCSS compared to non-RT groups [24]. However, the value of postoperative adjuvant radiotherapy for MPTB patients in different age groups has not been deeply studied. Therefore, this study focused on the effect of adjuvant radiotherapy on survival outcomes in MPTB patients of different ages to explore whether there are age subgroups that could benefit from radiotherapy. By analyzing the long-term survival outcomes of 2,261 MPTB patients diagnosed between 2000 and 2020 (including 465 patients treated with RT), patients who did not receive RT had better survival outcomes than those who received RT, both in terms of OS (*p* = 0.002) (Fig. 2A) and BCSS (*p* < 0.001) (Fig. 3A). Previous studies on MPTB using SEER program data (1983–2002) similarly showed that patients who received RT after surgery had more adverse survival outcomes compared with those who did not receive RT [8]. This is similar to the results of our study. 2261 patients with MPTB were divided into four subgroups according to age (18–45, 46–55, 56–65, 66–80), and no age group that could benefit from radiotherapy was found after analyzing the long-term survival outcomes (age group of 18–45 years: OS *P* = 0.019, BCSS *P* = 0.016; age group of 46–55 years: OS *P* < 0.001, BCSS *P* < 0.001; age group of 56–65 years: OS *P* = 0.685, BCSS *P* = 0.740; age group of 66–80 years: OS *P* = 0.658, BCSS *P* = 0.695, respectively) (Figs. 2, 3). After PSM, the results remain consistent (age group of 18–45 years: OS *P* = 0.473, BCSS *P* = 0.750; age group of 46–55 years: OS *P* = 0.380, BCSS *P* = 0.816; age group of 56–65 years: OS *P* = 0.484, BCSS *P* = 0.290; age group of 66–80 years: OS *P* = 0.997, BCSS *P* = 0.763, respectively) (Figs. 4 and 5). The multivariate Cox regression analysis in this study revealed (Tables 2 and S1) that there was no survival benefit from radiotherapy for patients with MPTB. A multicenter retrospective study concluded that patients with adverse prognostic factors, such as tumor necrosis or size, should be considered for RT [17]. However, this study only demonstrated the benefit of RT for local control of patients with MPTB and did not demonstrate the benefit of RT on long-term survival of patients with MPTB. Few studies have shown that increased local control rates in the RT group are associated with improved long-term survival outcomes. In conclusion, in this study, no age-specific subgroup of MPTB patients were found to benefit from RT in terms of long-term survival, which is consistent with previous findings.

There are several limitations to this study. First of all, borderline PTB may be incorrectly classified as MPTB in the SEER database, thereby affecting the results. Secondly, some patients’ information, including tumor grade, stage, lymph node status, and metastasis, was missing from the SEER database. Thirdly, the SEER database must also be continuously improved since it lacks information on local recurrence and histopathology, including the status of resection margins.

## Conclusions

Based on an analysis of the SEER database, no patients with MPTB in specific age groups were found to benefit from RT in terms of long-term survival. Therefore, a large number of studies are still needed to explore the subgroups of MPTB patients who could benefit from RT.

### Supplementary Information

**Additional file 1** **Table S1** Multivariate Cox regression model analysis of BCSS in different age groups

**Additional file 2** **Table S2** Characteristics of MPTB patients 1:1 matched Without vs With RT in different age groups

## Data Availability

The original data are available from the SEER database or on request from the corresponding author.

## Abbreviations

MPTB: Malignant phyllodes tumor of the breast
RT: Radiotherapy
OS: Overall survival
BCSS: Breast cancer-specific survival
PSM: Propensity score matching
