# Supplementary material for: Effect of adjuvant radiotherapy on overall survival and breast cancer-specific survival of patients with malignant phyllodes tumor of the breast in different age groups: a retrospective observational study based on SEER
Source: Radiat Oncol. 2024 May 21;19:59. doi: 10.1186/s13014-024-02442-5 (PMC11107058; doi:10.1186/s13014-024-02442-5)
Supplement: Supplementary file 1 — Additional file 1 Table S1 Multivariate Cox regression model analysis of BCSS in different age groups [file 13014_2024_2442_MOESM1_ESM.docx]

| Charactreristics | 18-45 |  | 46-55 |  | 56-65 |  | 66-80 |  |
| --- | --- | --- | --- | --- | --- | --- | --- | --- |
|  | HR(95%C) | P | HR(95%C)\ | P | HR(95%C) | P | HR(95%C) | P |
| **Year** |  |  |  |  |  |  |  |  |
| 2000-2009 | Reference |  | Reference |  | Reference |  | Reference |  |
| 2010-2020 | 45.776(5.739 - 365.099) | < 0.001 | 14.403(6.028– 34.415) | 0.275 | 0.682 (0.370 - 1.256) | 0.219 | 3.029 (1.282 - 7.158) | 0.012 |
| **Race** |  |  |  |  |  |  |  |  |
| White | Reference |  | Reference |  | Reference |  | Reference |  |
| Black | 0.605(0.210 - 1.747) | 0.353 | 0.942 (0.450 - 1.975) | 0.875 | 1.696 (0.731 - 3.934) | 0.219 | 1.529 (0.606 - 3.859) | 0.369 |
| Other | 1.086 (0.547 - 2.155) | 0.813 | 1.095 (0.629 - 1.904) | 0.748 | 0.567 (0.242 - 1.328) | 0.191 | 0.434 (0.140 - 1.348) | 0.149 |
| **Tumor grade** |  |  |  |  |  |  |  |  |
| I-II | Reference |  | Reference |  | Reference |  | Reference |  |
| III-IV | 1.417 (0.620 - 3.240) | 0.409 | 1.264 (0.752 - 2.124) | 0.377 | 3.032 (1.571 - 5.852) | < 0.001 | 1.074 (0.561 - 2.057) | 0.828 |
| unknown | 1.665 (0.812 - 3.416) | 0.164 | 0.710 (0.391 - 1.289) | 0.260 | 1.750 (0.852 - 3.594) | 0.127 | 0.859 (0.348 - 2.117) | 0.741 |
| **Laterality** |  |  |  |  |  |  |  |  |
| left | Reference |  | Reference |  | Reference |  | Reference |  |
| right | 0.735 (0.418 - 1.290) | 0.283 | 0.884 (0.577 - 1.355) | 0.572 | 0.839 (0.500 - 1.409) | 0.507 | 0.856 (0.486 - 1.509) | 0.591 |
| **AJCC.T** |  |  |  |  |  |  |  |  |
| T1-T2 | Reference |  | Reference |  | Reference |  | Reference |  |
| T3-T4 | 1.219 (0.636 - 2.334) | 0.551 | 1.235 (0.747 - 2.043) | 0.410 | 1.772 (0.956 - 3.285) | 0.069 | 1.602 (0.767 - 3.345) | 0.210 |
| unknown | 0.377 (0.058 - 2.435) | 0.306 | Inf | 0.995 | 0.704 (0.100 - 4.981) | 0.725 | 7.519 (2.651 - 21.329) | <0.001 |
| **AJCC.N** |  |  |  |  |  |  |  |  |
| negative | Reference |  | Reference |  | Reference |  | Reference |  |
| positive | 6.577 (1.370 - 31.584) | 0.019 | 0.000 (0.000 - Inf) | 0.999 | 6.913 (2.274 - 21.018) | <0.001 | 1.437 (0.166 - 12.459) | 0.742 |
| unknown | 0.109 (0.006 - 1.859) | 0.126 | 0.411 (0.119 - 1.417) | 0.159 | 1.589 (0.588 - 4.295) | 0.361 | 2.120 (0.758 - 5.930) | 0.152 |
| **AJCC.M** |  |  |  |  |  |  |  |  |
| negative | Reference |  | Reference |  | Reference |  | Reference |  |
| positive | 7.208 (0.445 - 116.759) | 0.165 | 0.000 (0.000 - Inf) | 0.996 | 15.972(4.622 - 55.188) | <0.001 | NA |  |
| unknown | 169.793(5.734-5027.813) | 0.003 | 0.000 (0.000 - Inf) | 0.996 | 5.369 (0.778 - 37.032) | 0.088 | NA |  |
| **Surgery of primary site** |  |  |  |  |  |  |  |  |
| BCS | Reference |  | Reference |  | Reference |  | Reference |  |
| mastectomy | 0.985 (0.525 - 1.846) | 0.962 | 0.666 (0.412 - 1.077)) | 0.098 | 2.148 (1.115 - 4.135) | 0.022 | 1.313 (0.675 - 2.557) | 0.423 |
| **Radiotherapy** |  |  |  |  |  |  |  |  |
| no | Reference |  | Reference |  | Reference |  | Reference |  |
| yes | 1.097 (0.504 - 2.386) | 0.816 | 1.294 (0.748 - 2.241) | 0.357 | 0.633 (0.319 - 1.258) | 0.192 | 1.024 (0.483 - 2.170) | 0.951 |
| **Chemotherapy** |  |  |  |  |  |  |  |  |
| no | Reference |  | Reference |  | Reference |  | Reference |  |
| yes | 0.505 (0.086 - 2.955) | 0.449 | 0.720 (0.170 - 3.042) | 0.655 | 0.928 (0.273 - 3.152) | 0.905 | 0.965 (0.202 - 4.608) | 0.965 |
| **Marital status** |  |  |  |  |  |  |  |  |
| married | Reference |  | Reference |  | Reference |  | Reference |  |
| unmarried | 0.851 (0.466 - 1.553) | 0.599 | 1.018 (0.638 - 1.623) | 0.941 | 0.712 (0.412 - 1.232) | 0.225 | 1.624 (0.924 - 2.855) | 0.092 |
| unknown | 0.230 (0.030 - 1.772) | 0.158 | 0.531 (0.189 - 1.489) | 0.229 | 0.470 (0.110 - 2.008) | 0.308 | 0.000 (0.000 - Inf) | 0.997 |
| **Local-lymphatic biopsy** |  |  |  |  |  |  |  |  |
| no | Reference |  | Reference |  | Reference |  | Reference |  |
| yes | 0.918 (0.438 - 1.925) | 0.821 | 0.892 (0.525 - 1.513) | 0.671 | 0.706 (0.394 - 1.266) | 0.243 | 0.505 (0.269 - 0.948) | 0.034 |
