# Supplementary material for: Effect of adjuvant radiotherapy on overall survival and breast cancer-specific survival of patients with malignant phyllodes tumor of the breast in different age groups: a retrospective observational study based on SEER
Source: Radiat Oncol. 2024 May 21;19:59. doi: 10.1186/s13014-024-02442-5 (PMC11107058; doi:10.1186/s13014-024-02442-5)
Supplement: Supplementary file 2 — Additional file 2 Table S2 Characteristics of MPTB patients 1:1 matched Without vs With RT in different age groups [file 13014_2024_2442_MOESM2_ESM.docx]

| **Characteristics** | **18~45 (n=252)** | | | **46~55**  **(n=294)** | | | **56~65**  **(n=216)** | | | **66~80**  **(n=96)** | | |
| --- | --- | --- | --- | --- | --- | --- | --- | --- | --- | --- | --- | --- |
|  | **RT**  **(n=126)** | **Non-RT**  **(n=126)** | ***P*** | **RT**  **(n=147)** | **Non-RT**  **(n=147)** | ***P*** | **RT**  **(n=108)** | **Non-RT**  **(n=108)** | ***P*** | **RT**  **(n=48)** | **Non-RT**  **(n=48)** | ***P*** |
| **Year** |  |  | 0.351 |  |  | 0.897 |  |  | 0.876 |  |  | 0.660 |
| 2000-2009 | 46 (36.5%) | 39 (31%) |  | 42 (28.6%) | 41 (27.9%) |  | 27 (25%) | 28 (25.9%) |  | 16 (33.3%) | 14 (29.2%) |  |
| 2010-2020 | 80 (63.5%) | 87 (69%) |  | 105 (71.4%) | 106 (72.1%) |  | 81 (75%) | 80 (74.1%) |  | 32 (66.7%) | 34 (70.8%) |  |
| **Race** |  |  | 0.964 |  |  | 0.964 |  |  | 0.961 |  |  | 0.561 |
| White | 86 (68.3%) | 84 (66.7%) |  | 104 (70.7%) | 106 (72.1%) |  | 82 (75.9%) | 82 (75.9%) |  | 36 (75%) | 35 (72.9%) |  |
| Black | 16 (12.7%) | 17 (13.5%) |  | 14 (9.5%) | 13 (8.8%) |  | 10 (9.3%) | 11 (10.2%) |  | 4 (8.3%) | 2 (4.2%) |  |
| Others | 24 (19%) | 25 (19.8%) |  | 29 (19.7%) | 28 (19%) |  | 16 (14.8%) | 15 (13.9%) |  | 8 (16.7%) | 11 (22.9%) |  |
| **Tumor grade** |  |  | 0.730 |  |  | 0.886 |  |  | 0.939 |  |  | 0.358 |
| Unknown | 42 (33.3%) | 39 (31%) |  | 30 (20.4%) | 29 (19.7%) |  | 33 (30.6%) | 31 (28.7%) |  | 11 (22.9%) | 6 (12.5%) |  |
| I-II | 41 (32.5%) | 47 (37.3%) |  | 49 (33.3%) | 53 (36.1%) |  | 44 (40.7%) | 44 (40.7%) |  | 18 (37.5%) | 18 (37.5%) |  |
| III-IV | 43 (34.1%) | 40 (31.7%) |  | 68 (46.3%) | 65 (44.2%) |  | 31 (28.7%) | 33 (30.6%) |  | 19 (39.6%) | 24 (50%) |  |
| **Laterality** |  |  | 0.801 |  |  | 0.641 |  |  | 0.785 |  |  | 0.414 |
| Left | 67 (53.2%) | 65 (51.6%) |  | 73 (49.7%) | 69 (46.9%) |  | 58 (53.7%) | 60 (55.6%) |  | 27 (56.2%) | 23 (47.9%) |  |
| Right | 59 (46.8%) | 61 (48.4%) |  | 74 (50.3%) | 78 (53.1%) |  | 50 (46.3%) | 48 (44.4%) |  | 21 (43.8%) | 25 (52.1%) |  |
| **AJCC-T** |  |  | 0.730 |  |  | 0.528 |  |  | 0.469 |  |  | 0.973 |
| Unknown | 29 (23%) | 33 (26.2%) |  | 36 (24.5%) | 41 (27.9%) |  | 23 (21.3%) | 19 (17.6%) |  | 11 (22.9%) | 11 (22.9%) |  |
| T1-T2 | 37 (29.4%) | 32 (25.4%) |  | 40 (27.2%) | 32 (21.8%) |  | 37 (34.3%) | 32 (29.6%) |  | 18 (37.5%) | 17 (35.4%) |  |
| T3-T4 | 60 (47.6%) | 61 (48.4%) |  | 71 (48.3%) | 74 (50.3%) |  | 48 (44.4%) | 57 (52.8%) |  | 19 (39.6%) | 20 (41.7%) |  |
| **AJCC-N** |  |  | 0.759 |  |  | 0.597 |  |  | 0.834 |  |  | 0.842 |
| Unknown | 27 (21.4%) | 31 (24.6%) |  | 4 (2.7%) | 7 (4.8%) |  | 4 (3.7%) | 3 (2.8%) |  | 1 (2.1%) | 1 (2.1%) |  |
| Negative | 96 (76.2%) | 91 (72.2%) |  | 140 (95.2%) | 138 (93.9%) |  | 101 (93.5%) | 103 (95.4%) |  | 45 (93.8%) | 46 (95.8%) |  |
| Positive | 3 (2.4%) | 4 (3.2%) |  | 3 (2%) | 2 (1.4%) |  | 3 (2.8%) | 2 (1.9%) |  | 2 (4.2%) | 1 (2.1%) |  |
| **AJCC-M** |  |  | 0.836 |  |  | 0.778 |  |  | 0.722 |  |  | 1.000 |
| Unknown | 27 (21.4%) | 31 (24.6%) |  | 35 (23.8%) | 40 (27.2%) |  | 22 (20.4%) | 18 (16.7%) |  | 11 (22.9%) | 11 (22.9%) |  |
| Negative | 98 (77.8%) | 94 (74.6%) |  | 106 (72.1%) | 102 (69.4%) |  | 84 (77.8%) | 87 (80.6%) |  | 36 (75%) | 36 (75%) |  |
| Positive | 1 (0.8%) | 1 (0.8%) |  | 6 (4.1%) | 5 (3.4%) |  | 2 (1.9%) | 3 (2.8%) |  | 1 (2.1%) | 1 (2.1%) |  |
| **Surgery of primary site** |  |  | 0.447 |  |  | 0.907 |  |  | 0.586 |  |  | 1.000 |
| Breast-conserving surgery | 59 (46.8%) | 53 (42.1%) |  | 72 (49%) | 73 (49.7%) |  | 53 (49.1%) | 49 (45.4%) |  | 24 (50%) | 24 (50%) |  |
| Mastectomy | 67 (53.2%) | 73 (57.9%) |  | 75 (51%) | 74 (50.3%) |  | 55 (50.9%) | 59 (54.6%) |  | 24 (50%) | 24 (50%) |  |
| **Chemotherapy** |  |  | 0.625 |  |  | 0.375 |  |  | 0.235 |  |  | 1.000 |
| No | 116 (92.1%) | 118 (93.7%) |  | 134 (91.2%) | 138 (93.9%) |  | 100 (92.6%) | 104 (96.3%) |  | 47 (97.9%) | 46 (95.8%) |  |
| Yes | 10 (7.9%) | 8 (6.3%) |  | 13 (8.8%) | 9 (6.1%) |  | 8 (7.4%) | 4 (3.7%) |  | 1 (2.1%) | 2 (4.2%) |  |
| **Marital status** |  |  | 0.492 |  |  | 0.249 |  |  | 0.393 |  |  | 0.834 |
| Unknown | 8 (6.3%) | 4 (3.2%) |  | 7 (4.8%) | 4 (2.7%) |  | 4 (3.7%) | 1 (0.9%) |  |  |  |  |
| Married | 61 (48.4%) | 62 (49.2%) |  | 82 (55.8%) | 72 (49%) |  | 65 (60.2%) | 68 (63%) |  | 29 (60.4%) | 30 (62.5%) |  |
| Not-married | 57 (45.2%) | 60 (47.6%) |  | 58 (39.5%) | 71 (48.3%) |  | 39 (36.1%) | 39 (36.1%) |  | 19 (39.6%) | 18 (37.5%) |  |
| **Local lymphatic biopsy** |  |  | 0.892 |  |  | 0.603 |  |  | 0.881 |  |  | 0.660 |
| Yes | 40 (31.7%) | 39 (31%) |  | 43 (29.3%) | 39 (26.5%) |  | 32 (29.6%) | 31 (28.7%) |  | 16 (33.3%) | 14 (29.2%) |  |
| No | 86 (68.3%) | 87 (69%) |  | 104 (70.7%) | 108 (73.5%) |  | 76 (70.4%) | 77 (71.3%) |  | 32 (66.7%) | 34 (70.8%) |  |
